# Enhancing the purity and intrinsic properties of ovine dermal papilla cells through flow cytometry sorting and cellular interactions

**DOI:** 10.5713/ab.24.0805

**Published:** 2025-04-01

**Authors:** Yuan Gou, Tingyan Hu, Lijuan Zhu, Xiaoyang Lv, Yutao Li, Rui Su, Zhenghai Song, Shanhe Wang, Wei Sun

**Affiliations:** 1 College of Animal Science and Technology, Yangzhou University, Yangzhou, China; 2 Joint International Research Laboratory of Agriculture and Agri-Product Safety, Ministry of Education of China, Yangzhou University, Yangzhou, China; 3 International Joint Research Laboratory in Universities of Jiangsu Province of China for Domestic Animal Germplasm Resources and Genetic Improvement, Yangzhou University, Yangzhou, China; 4 CSIRO Agriculture and Food, Brisbane, Australia; 5 Suzhou Sheep Breeding Farm, Suzhou City, Jiangsu, China; 6 Dongshan Animal Epidemic Prevention Station, Suzhou, China

**Keywords:** Cellular Interactions, Dermal Papilla Cells (DPCs), Flow Cytometry, Hair Follicle, PDGFRA

## Abstract

**Objective:**

Dermal Papilla Cells (DPCs) play a crucial role in regulating hair follicle development and serve as a valuable *in vitro* model for screening and analyzing the genes associated with this process. However, current methods for isolating ovine DPCs primarily rely on mechanical techniques, which present several limitations. The aim of this study is to establish a method for isolating and culturing ovine DPCs with high purity and retaining their intrinsic properties.

**Methods:**

We identified sheep DPC membrane-specific genes using single-cell transcriptomic data, validated by immunostaining and flow cytometry. Antibody-labeled DPCs were isolated, cultured, and assessed via fluorescence-activated cell sorting (FACS), comparing their purity with conventional mechanical isolation. Mechanically isolated and flow-sorted DPCs were analyzed through agglutination, cell counting kit (CCK-8), and EDU staining. Furthermore, we examined the biological properties of isolated DPCs in conditioned media using CCK-8, EDU, and quantitative reverse transcription polymerase chain reaction assays.

**Results:**

PDGFRA was identified as a marker for ovine DPCs. Flow cytometry showed that PDGFRA-labeled DPCs made up 1.54% of the hair follicle cell population, with 1.92% live DPCs obtained via FACS. The isolated DPCs demonstrated agglutination and were positive for ALP, Versican, and α-SMA. Antibody labeling yielded higher DPC purity compared to mechanical isolation, highlighting its efficiency. Accordingly, the addition of conditioned media from mechanically isolated DPCs significantly enhanced agglutination, cell viability, proliferation, and inductive capacity of the sorted DPCs. However, as the number of passages increased, the sorted DPCs demonstrated significant disadvantages in cell agglutination, proliferation rate, and viability compared to mechanically isolated DPCs. Accordingly, the addition of conditioned media from mechanically isolated DPCs significantly enhanced agglutination, cell viability, proliferation, and inductive capacity of the sorted DPCs.

**Conclusion:**

This study highlights the effectiveness of antibody labeling and flow cytometry for isolating functionally pure DPCs, as well as the potential of conditioned media to maintain the functional properties of these cells.

## INTRODUCTION

Wool is a valuable textile raw material with significant economic importance, and the morphogenesis and development of hair follicles are key determinants of wool quality. Dermal papilla cells (DPCs), a specialized group of mesenchymal cells located at the base of hair follicles, are essential for initiating hair follicle formation and regulating their cyclical growth, directly influencing wool production and quality [1,2]. The ability to induce hair follicle formation is the most criticalfunctional property of DPCs [3,4]. Cohen J’s study confirmed that intact DPCs possess the ability to induce hair follicle regeneration. Additionally, Jahoda was the first to demonstrate that rat vibrissa DPCs, even when cultured *in vitro*, retain this regenerative capability, highlighting the functional potential of DPCs in hair follicle development [5,6].

Based on these properties, DPCs serve as an essential model for screening and identifying functional genes involved in hair follicle development *in vitro*. Furthermore, they act as seed cells for constructing hair follicle organoids or inducing hair follicle regeneration. Obtaining a large quantity of DPCs is crucial for studying their properties and functions, advancing molecular breeding in ovine, and improving the quality of ovine wool.

Currently, the primary methods for isolating DPCs *in vitro* include: 1) the surgical microdissection method [7–9], in which DPCs are obtained by isolating individual hair follicles using instruments such as scalpels, tweezers, and microscopes [9]. Microdissection is the main method for isolating DPCs. However, obtaining DPCs using this method is inefficient. Not only is this method time-consuming and labor-intensive, but the isolated DPCs are also less pure and often mixed with other nearby cells, making them difficult to distinguish in culture. Additionally, the number of DPCs obtained through isolation is small. Typically, this method is only effective for isolating larger hair follicles, such as those in the human scalp and mouse vibrissae [10,11]. Smaller follicles, such as those on the backs of mice, are more challenging and time-consuming to handle, with a higher risk of cell contamination. Although some researchers have improved microdissection methods by combining them with enzymatic digestion to shorten operation time, challenges remain [12,13], DPCs obtained through this method still encounter issues, such as poor adhesion to culture surfaces and slow growth. 2) Genetically labeled DPCs: This method involves using genes specifically expressed in DPCs as promoters to construct transgenic mice. Fluorescent proteins are then expressed in DPCs, allowing viable cells to be sorted using flow cytometry. While effective, constructing transgenic animals is costly and time-consuming. Current successful transgenic models, such as those using Versican or Lef1, enable DPCs to express fluorescent proteins, facilitating easier flow sorting [14]. 3) Antibody labeling method [15], The antibody labeling method uses specific surface antibodies to label DPCs, enabling large-scale sorting with a flow sorter. The primary limitation of this method is the challenge of identifying surface antibodies with high specificity for DPCs. Some studies have successfully used prominin-1 (CD133) to isolate mouse DPCs from skin. However, as mice age, the expression of prominin-1 (CD133) declines sharply, making it unsuitable for isolating DPCs from adult mice. Another study reported that antibodies targeting the LEPR receptor and Scavenger Receptor Class A Member 5 can be used to sort DPCs during the growth phase in mice [16].

Currently, ovine DPCs are primarily isolated through microdissection and enzymatic digestion. However, thislack of consensus on DPC morphology, accurately identifying mechanically isolated DPCs remains challenging, and contamination by during the culture process. Furthermore, because ovine DPC membrane molecular markers have not been systematically characterized, there are few reports on using antibody markers for ovine DPC isolation. As a result, current methods for isolating ovine DPCs remain limited and are unable to meet the demands for general, large-scale, or specialized separations. Previous single-cell sequencing studies have laid the groundwork for screening membrane-specific genes in DPCs [17].

DPCs exist within a three-dimensional, onion-like structure *in vivo*. They are encapsulated by the hair matrix on the upper side and surrounded by cells from the dermal sheath cup at the lower part, creating a dynamic niche that facilitates bidirectional interactions with neighboring cells, essential for regulating hair follicle development and regeneration [18]. This microenvironment encompasses not only the extracellular matrix and small molecules but also various adjacent cells and their spatial relationships [19]. The bidirectional communication between DPCs and their microenvironment is crucial for regulating processes such as cell proliferation, metabolism, and the activation of polar transcription factors. Therefore, maintaining the properties of DPCs in culture requires careful consideration of their interactions with surrounding cells. However, many studies have been limited by neglecting the comprehensive role of the DPC microenvironment, potentially impacting the functionality and stability of cultured DPCs.

The aim of this study was to obtain ovine DPCs with high purity and distinct properties. PDGFRA was successfully identified as a specific surface marker for DPCs in hair follicles by immunofluorescence and flow cytometry. Using this marker, flow cytometry efficiently isolated DPCs labeled with PDGFRA. In addition, comparative analyses of mechanically separated DPCs and flow-sorted DPCs were performed to assess the differences in their properties. Finally, the intrinsic properties of late-stage DPCs after sorting were enhanced according to cellular interactions.

## MATERIALS AND METHODS

### Animals

All experimental procedures and the use of ovine in this study were pre-approved by the Experimental Animal Management Committee of Yangzhou University (Approval No. NSFC2020 NFY-1).

### Collection of sheep skin samples

Skin tissues from Hu sheep were obtained from Suzhou Sheep Farm. Three healthy, three-day-old lambs (one male and two females) were anesthetized by subcutaneous injection of 2% lidocaine hydrochloride (10 mg/kg). Skin samples, approximately 1 cm^2^ in size and 2 mm in depth, were collected. The samples were divided into two parts: one part was stored in Dulbecco’s Modified Eagle Medium (DMEM)/F12 (Thermo Fisher Scientific, Waltham, MA, USA) with penicillin-streptomycin for cell culture, and the other was fixed in 4% paraformaldehyde (Solarbio, Beijing, China) for further analysis. Following sample collection, the wounds were sutured to ensure proper healing.

### Skin embedding and immunostaining

Skin samples fixed in paraformaldehyde were dehydrated through a graded ethanol series (50%, 70%, 80%, 95%), washed with xylene for 30 minutes, and embedded in paraffin. Sections were cut into 5 μm slices using a Leica RM2255 microtome (Leica Biosystems, Nussloch, Germany), floated on 45°C water, mounted on slides, dried, and trimmed. Before immunofluorescence staining, sections were dehydrated twice in xylene for 30 minutes each, then rehydrated through a graded ethanol series (100%, 95%, 85%, 70%, 50%) and ultrapure water for 3 minutes each. Antigen retrieval was performed by heating the sections in sodium citrate buffer at 96°C for 10 minutes. The sections were then blocked in 10% goat serum and 3% bovine serum albumin (Merck KGaA, Darmstadt, Germany) in phosphate-buffered saline (PBS) for 40 minutes at room temperature, followed by overnight incubation with primary antibodies at 4°C. Fluorescently labeled secondary antibodies were applied for 30 minutes at 37°C. Nuclei were counterstained with Hoechst 33342 (Beyotime Biotechnology, Shanghai, China), and sections were sealed with VECTASHIELD solid mounting medium (Vector, Torrance, CA, USA). Each section was prepared with three biological replicates, and fluorescence images were captured using a confocal microscope.

### Preparation of hair follicle cell suspension from ovine back skin

Freshly excised sheep back skin samples (approximately 1×1 cm^2^) were rinsed three times (5 min each) in pre-cooled PBS with gentle agitation to remove surface debris. The epidermis was then mechanically separated from the dermis with a sterile scalpel blade in DMEM supplemented with 2% penicillin-streptomycin, and all operations were performed on ice to minimize cellular stress. Intact follicular units were carefully extracted with fine forceps, and hair follicles were placed in 0.25% type IV collagenase (C8160; Solarbio) and digested for 60 min at 37°C, using an orbital shaker at 100 rpm and gently pipetted every 15 min. The collagenase was carefully aspirated off by centrifugation (10×g, 5 min) and the tissue pellet was treated with 0.25% trypsin-EDTA (T1320; Solarbio) for 30 min at 37°C before digestion was stopped by the addition of DMEM containing 10% FBS. The cell suspension was immediately placed on ice and filtered sequentially through 100 μm and 40 μm nylon mesh to remove undigested aggregates, followed by centrifugation (10×g, 10 min). The resulting precipitate was resuspended in DMEM medium supplemented with 10% FBS.

### Analysis of PDGFRA^+^ dermal papilla cell rate by flow cytometry

The proportion of PDGFRA^+^ DPCs in hair follicles was quantified by fluorescence-activated cell sorting (FACS) LSRFortessa (BD Biosciences, San Jose, CA, USA). Briefly, single-cell suspensions were adjusted to 1×10^6^ cells/mL in PBS containing 2% FBS. Cells were incubated with a 1:100 dilution of anti-PDGFRA monoclonal antibody (ab270086; Abcam, Cambridge, UK) at 4°C in the dark for 40 min. After staining, cells were washed twice with PBS and then resuspended in 500 μL PBS. Data were acquired using a 488 nm excitation emission filter. Gating was performed based on forward scatter - area (FSC-A) and side-scatter area parameters, and debris was excluded by double-resolving FSC-A vs. FSC-H. PE+ cells were identified using a threshold determined by isotype controls. All data were analyzed using FlowJo 10.0.7, and results are expressed as the percentage of PDGFRA^+^ cells.

### Dermal papilla cells live cell sorting

**Figure 1:**
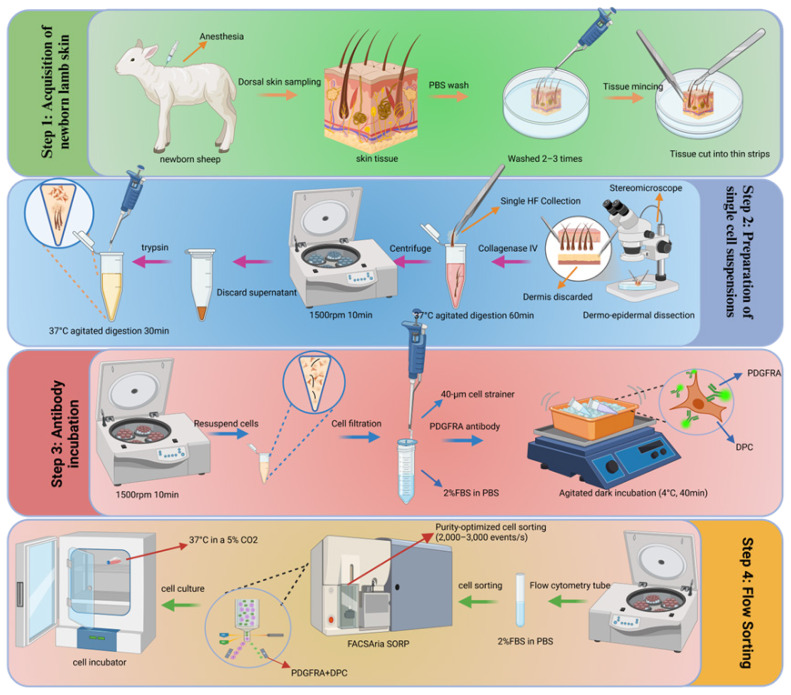
Experimental procedures for the sorting of sheep DPCs. DPCs, dermal papilla cells.

Freshly prepared single-cell suspensions were incubated with phycoerythrin (PE)-conjugated anti-PDGFRA antibody (1:100 dilution, ab270086; Abcam) for 30 min at 4°C in the dark, and then washed twice with PBS containing 2% fetal bovine serum (FBS). PE-labeled DPC were sorted using a FACSAria SORP flow cytometer (BD Biosciences) equipped with an 85 μm nozzle and 45 psi sheath pressure. To ensure high purity, cells were sorted at 2,000 to 3,000 events per second in purity optimization mode. Sorting strategies included (1) debris exclusion by forward and side scatter, (2) double acrosome discrimination using FSC-A versus FSC-H parameters, and (3) identification of PE^+^ cells based on fluorescence thresholds determined from unstained controls. Sorted DPCs were collected in DMEM medium supplemented with 20% FBS and data analysis was performed using FlowJo 10.0.7. Sorted DPCs were cultured in DMEM/F12 medium (HyClone, Cytiva, Logan, UT, USA) supplemented with 10% FBS (Gibco, Waltham, MA, USA) and 1% penicillin-streptomycin (Gibco) at 37°C in a 5% CO_2_ environment. In summary, the complete process of hair papilla cell sorting is shown in the diagram (Figure 1).

### Dermal papilla cells mechanical separation and purification

Referring to Rufaut’s method [20], the skin tissue obtained was separated from the loose subcutaneous connective tissue, washed three times with PBS containing 1% penicillin and streptomycin, and cut into strips. Hair follicles were extracted into 24-well plates under a microscope, and the hair papillae were punctured with a 1 ml syringe needle to allow the outflow of DPCs, and the growth of native DPCs was observed after 5 to 7 days. A significant slowdown in DPC growth could be observed after two weeks of incubation, at which point the first purification after isolation was performed. Each well was digested with 100 μL of 0.25% trypsin preheated at 37°C for 2 min at room temperature, the digest was discarded and washed with PBS containing 1% penicillin-streptomycinfor 10s. PBS was discarded and 100 μL of 0.25% trypsin was added to each well. Discard PBS and add 100 μL of 0.25% trypsin pre-warmed at 37°C to each well and digest for 8 min at room temperature. 400 μL of complete medium was added to each well to terminate the digestion and the cells were gently pipetted gently to completely isolate the cells into a single cell state. The cell suspension was sieved through a cell sieve and collected into a 2 mL sterile centrifuge tube and centrifuged at 10×g for 8 min. After centrifugation, a very small amount of precipitate can be seen at the bottom of the centrifuge tube. After carefully discarding the supernatant, the cells were resuspended by adding 1 mL of PBS, and 2 to 3 tubes of cells were pooled together in one centrifuge tube and centrifuged at 10×g for 5 min. Discard PBS, add 1 mL of complete medium to each tube to resuspend the cells, inoculate them homogeneously into 12-well cell culture plates, and continue to incubate in the incubator until the cells are completely confluent for the second purification.

### Cell slide immunofluorescence

Place the slides in a 24-well plate and seeded the hair follicle single-cell suspension and mechanically isolated DPCs onto separate slides. After the cells have proliferated, wash them with PBS and fix them with 4% paraformaldehyde for 30 minutes, followed by another PBS wash. Block the cells with a blocking solution for 30 minutes at room temperature. Transfer the slides, apply the primary antibody, and incubate for 2 hours. After incubation, wash the slides and apply the secondary antibody, incubating for 40 minutes. Add 4′,6-diamidino-2-phenylindole (DAPI) for counterstained, wash, and mount the slides using an antifade agent. Seal the slides with nail polish and observe under a fluorescence microscope. The antibodies used for immunofluorescence are listed in Table 1.

### Alkaline phosphatase staining

Approximately 5000 sorted cells were seeded into a 24-well plate and incubated. After sufficient proliferation, the culture medium was removed, and the wells were washed with PBS. The cells were then fixed with 4% paraformaldehyde at room temperature for 30 minutes, followed by three 10-minute washes with PBS. Subsequently, the cells were stained with a pre-prepared solution for 1 hour and washed three times with PBS for 5 minutes each. Brightfield images were captured using an inverted microscope.

### Conditioned media collection and cell culture

After purification of mechanically isolated DPCs to the third generation, the conditioned medium of the third generation of mechanically isolated DPCs was collected after 36 h of culture and set aside. The sorted DPCs were passaged to the eighth generation, and then washed with PBS containing 1% double antibody for 10 s. Discard the PBS, add 100 μL of preheated 0.25% trypsin to each well, and digest for 3 min at 37°C in a cell culture incubator, and then observe under an inverted microscope to see that most of the cells were digested to a state of suspension. Add 400 μL of complete medium to the culture flask to terminate the digestion and transfer to a 2 mL centrifuge tube and centrifuge at 10×g for 8 min. The centrifuged cells were spread evenly in a 12-well plate, and the pre-conditioned medium was added after 24 h. The cells were then transferred to a 12-well plate.

### CCK-8 assay

DPCs from both the sorted and mechanically isolated groups were seeded into 96-well plates at a density of 5,000 cells per well. Cell viability was assessed at 24 and 48 hours using the CCK-8 kit (Vazyme, Nanjing, China) according to the manufacturer’s instructions. Absorbance was measured at 450 nm with a multimodal microplate detection system (EnSpire, PerkinElmer, Waltham, MA, USA).

### EdU Assay

For the EdU assay, DPCs were cultured in 24-well plates. When the cell density reached 70%, the cells were labeled and stained using the EdU Apollo *in vitro* imaging kit (RiboBio, Guangzhou, China) according to the manufacturer’s instructions. After staining, the cells were observed and imaged under a fluorescence inverted microscope (DMi8; Leica). Three random areas were selected for analysis, and the number of stained cells was quantified using Image Pro Plus 6.0 software (Media Cybernetics, Rockville, MD, USA).

### Primers for quantitative reverse transcription polymerase chain reaction

In this study, primers for the relevant genes were designed using Primer Premier 5.0 software (Premier Biosoft International, Palo Alto, CA, USA). The primers were synthesized by Tsingke Biotechnology (Nanjing, China) and are listed in Table 2.

### Statistical analysis

Statistical analysis was conducted using SPSS 25.0 software (SPSS Inc., Chicago, IL, USA). An unpaired Student’s t-test was used for two-group comparisons. Statistical significance was defined as p<0.05(*), p<0.01 (**), or p<0.001 (***). Each assay in this study was performed in triplicate. All data are presented as the mean±standard error of the mean.

## RESULTS

### The PDGFRA antibody is specifically expressed on the cell membrane of dermal papilla cells and serves as an effective marker for flow sorting

**Figure 2:**
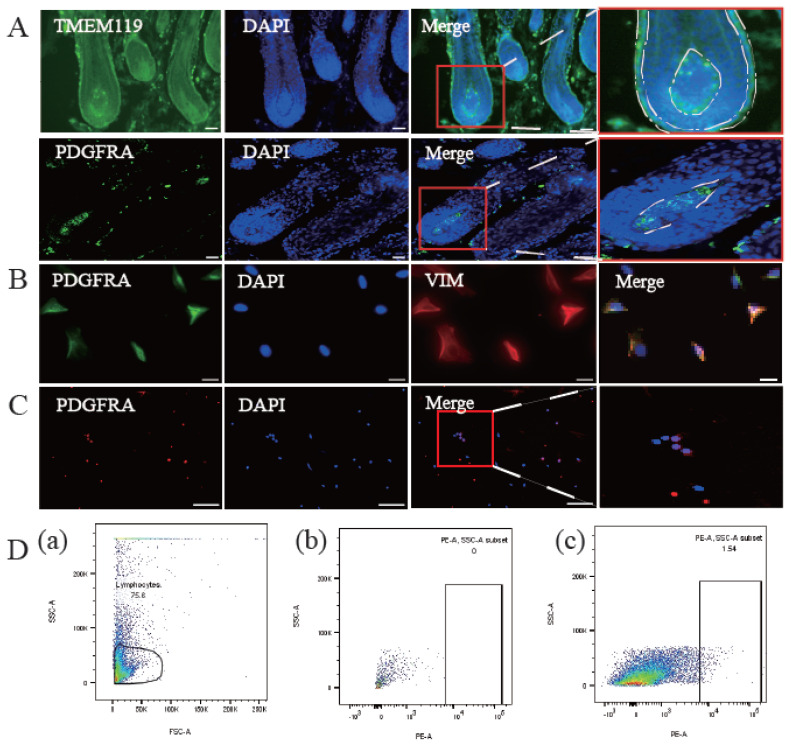
Screening and identification of surface proteins specifically expressed by DPCs. (A) Immunostaining of growing ovine dorsal skin was performed using PDGFRA and TMEM119 antibodies, with nuclei stained blue by DAPI and cells labeled green by PDGFRA and TMEM119 antibodies. Scale bar: 50 μm. (B) Immunostaining of mechanically isolated P3 DPCs using the PDGFRA antibody showed nuclei stained blue by DAPI, DPCs labeled green by PDGFRA, and cells marked red by VIM. Scale bar: 200 μm. (C) Immunofluorescence staining of hair follicle single-cell suspensions was conducted, with nuclei stained blue by DAPI and cells labeled red by the PDGFRA antibody. Scale bar: 100 μm. (D): (a) Circle gate used for PDGFRA labeling analysis of hair follicle single-cell suspensions. (b) Hair follicle cell suspensions not incubated with the PDGFRA antibody served as a negative control. (c) PDGFRA^+^ DPCs comprised 1.54% of the total hair follicle cell population. SSC-A, side scatter-area; PE, phycoerythrin; DAPI, 4′,6-diamidino-2-phenylindole; DPCs, dermal papilla cells.

Based on our previous single-cell transcriptome sequencing of lamb skin [17]. we screened for molecular markers specific to DPC membranes (Table 3), focusing on markers that met essential criteria, such as commercial antibody availability, high expression, conservation, and absence in keratinocytes. This screening identified PDGFRA and TMEM119 as potential candidates. Immunostaining of ovine dorsal skin revealed that PDGFRA was specifically expressed in DPCs, while TMEM119 lacked this specificity (Figure 2A). Further immunofluorescence staining of isolated P3 DPCs confirmed PDGFRA’s membrane localization (Figure 2B). Immunostaining of heterogeneous hair follicle cells showed the presence of a percentage of PDGFRA-labelled DPC (Figure 2C), whereas FACS LSRFortessa analysis showed 1.54% PDGFRA^+^DPC (Figure 2D), reinforcing its suitability for DPC sorting.

### Successful sorting of 1.92% of living dermal papilla cells based on flow cytometry combined with PDGFRA

**Figure 3:**
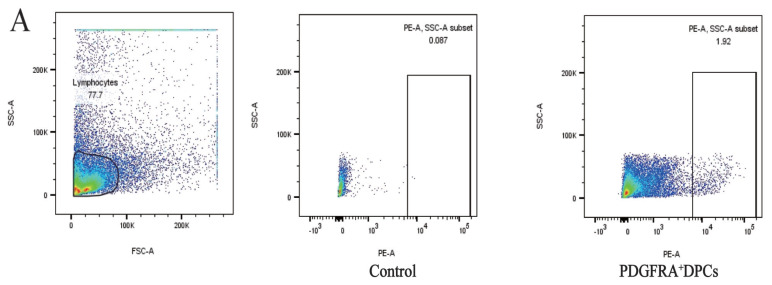
Flow cytometry-based sorting of living DPCs using PDGFRA antibody. (a) The gating strategy for the hair follicle single-cell suspension is illustrated in panel. (b) Shows Hair Follicle Cells that were not incubated with the PDGFRA antibody, serving as a negative control. (c) The sorted PDGFRA^+^ DPCs account for 1.92% of the total hair follicle cells. These results demonstrate the effectiveness of the PDGFRA antibody in specifically labeling and sorting DPCs. SSC-A, side scatter-area; FSC-A, forward scatter - area; PE, phycoerythrin; DPCs, dermal papilla cells.

Immunofluorescence and flow cytometry validated PDGFRA as an effective marker for sorting live DPCs. Ovine dorsal hair follicles were isolated, dissociated, and labeled with PDGFRA antibodies for flow cytometric analysis. During sorting, debris and cell aggregates were excluded, and PE^+^ cells—representing DPCs—constituted 1.92% of the total hair follicle cell population (Figure 3), consistent with previous findings.

### Sorted dermal papilla cells exhibit the correct identity and properties

**Figure 4:**
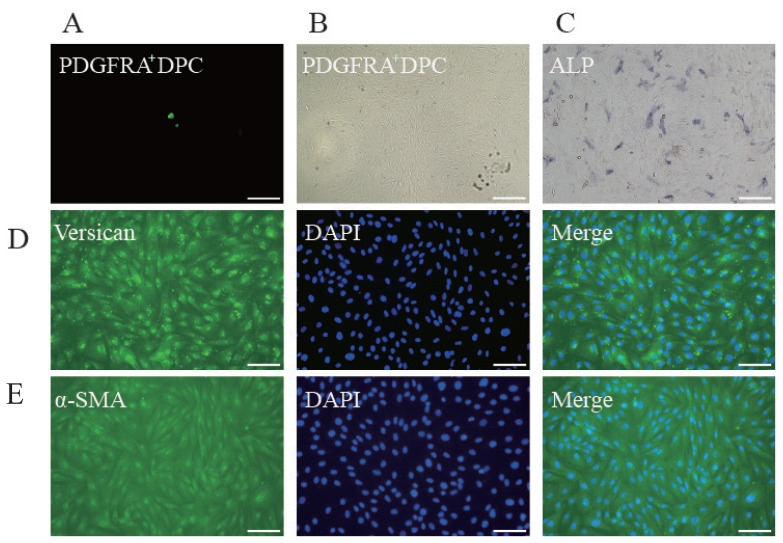
Culture and identification of DPCs after sorting. (A,B) P2 PDGFRA^+^ DPCs cultured *in vitro*. (C) P2 PDGFRA^+^ DPCs exhibit strong ALP activity. (D,E) Immunostaining for Versican and α-SMA, showing high positivity in PDGFRA^+^ DPCs. The scale bar in the figure is 100 μm. DAPI, 4′,6-diamidino-2-phenylindole; DPCs, dermal papilla cells.

After sorting, the PDGFRA^+^ cells retained their fluorescence, as confirmed by microscopy. To verify the accuracy of cell isolation, we assessed their properties. Given the limited number of sorted DPCs, we cultured and expanded them. Under the microscope, the sorted cells exhibited a flat, spindle-like morphology and demonstrated agglutination capacity (Figures 4A, 4B). Additionally, these DPCs showed alkaline phosphatase activity (Figure 4C). To further confirm their identity, we performed immunofluorescence staining for Versican and α-SMA, established molecular markers of DPCs. The results indicated strong marker activity in the stained DPCs (Figures 4D, 4E). These findings validate that the sorted cells were indeed DPCs and possessed characteristic DPC properties.

### Flow cytometry demonstrates that sorted dermal papilla cells are much purer than mechanically separated dermal papilla cells

**Figure 5:**
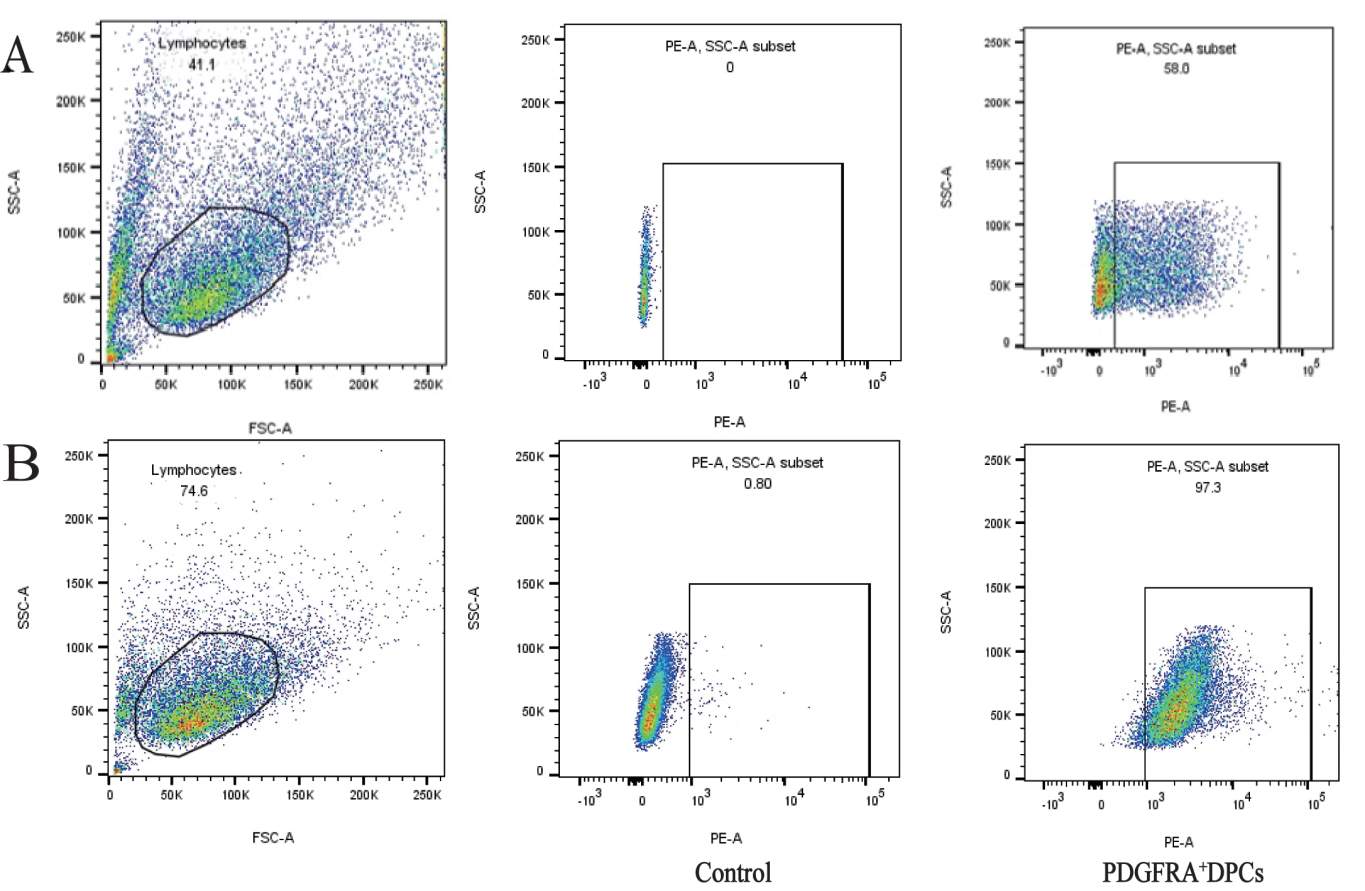
Flow cytometric analysis of mechanically isolated and antibody-labeled P3 DPCs using PDGFRA labeling. (A) PDGFRA^+^ DPCs obtained by mechanical isolation accounted for 58% of the total number of DPCs. (B) The positivity rate of DPCs obtained by antibody labeling was 97.3%. A round gate was used for the DPCs single-cell suspension. DPCs not incubated with the PDGFRA antibody served as a negative control. SSC-A, side scatter-area; PE, phycoerythrin; FSC-A, forward scatter - area; DAPI, 4′,6-diamidino-2-phenylindole; DPCs, dermal papilla cells.

To illustrate the advantages of antibody labeling over mechanical separation, we cultured DPCs obtained through both methods to passage 3 (P3) and assessed their purity using PDGFRA and flow cytometry. The results indicated that mechanical dissociation achieved a DPC purity of approximately 58% (Figure 5A), whereas antibody labeling yielded a significantly higher purity of 97.3% (Figure 5B), demonstrating a substantial improvement.

### Sorted dermal papilla cells are more susceptible to loss of properties than mechanically separated dermal papilla cells

**Figure 6:**
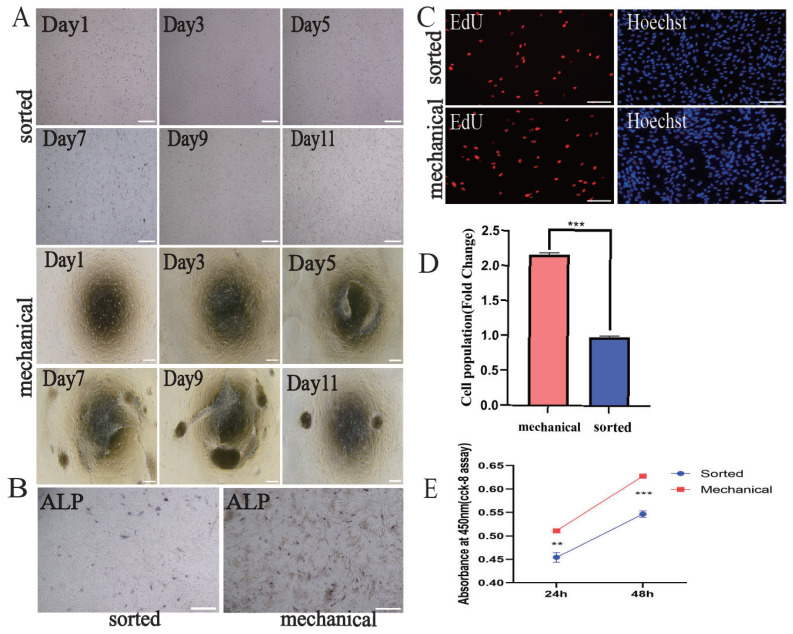
Analysis of DPC properties from mechanically isolated and flow-sorted cells. (A) P5 Mechanically separated DPCs were more capable of agglutinating after the fifth day than mechanically separated DPCs. (B) Mechanically separated DPC ALP is more active than fractionated DPC. (C) The proliferation of mechanically isolated DPCs was significantly higher than that of sorted DPCs as illustrated by EDU assay. (D) Shows the proportion of positive DPCs for each isolation method. (E) CCK-8 showed significantly higher activity of mechanically isolated DPC than sorted DPC at 24 h and 48 h. Data are mean±SEM (n = 3); significance determined by unpaired Student’s t-test (* p<0.05; ** p<0.01; *** p<0.001). Scale bars: 200 μm for A, B, C; 100 μm for D. DPCs, dermal papilla cells; SEM, standard error of the mean.

Sorted and mechanically isolated P5 DPCs were seeded into 6-well plates at a density of 1×10^6^ cells to observe growth characteristics. During the first 3 days, cells from both isolation methods expanded rapidly, filling the wells. However, by day 5, differences emerged: mechanically isolated DPCs continued to proliferate, forming clusters, while sorted DPCs began to show signs of cell death. By days 7, 9, and 11, this disparity became more pronounced; sorted DPCs were restricted to planar growth and failed to form clusters, even with media changes or subculturing (Figure 6A). Alkaline phosphatase results indicated that mechanically isolated DPCs exhibited greater positivity than sorted DPCs (Figure 6B). Similarly, EdU proliferation assays showed significantly higher levels in mechanically isolated DPCs compared to sorted DPCs (Figures 6C, 6D). Finally, the CCK-8 assay further confirmed the superior viability of mechanically isolated DPCs over sorted DPCs.

### Addition of conditioned media enhances dermal papilla cell properties

**Figure 7:**
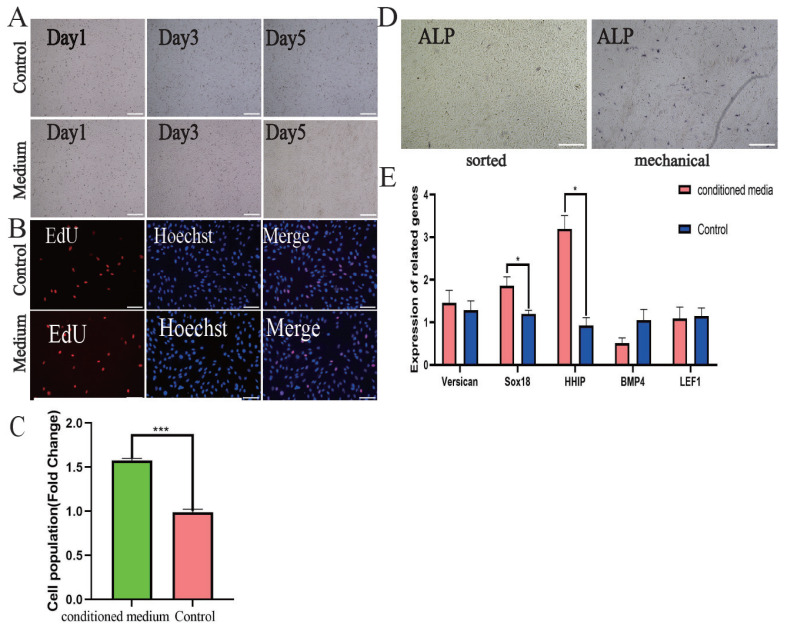
The effect of conditioned media on DPC properties was assessed. (A) Tendency of DPCs to agglutinate after addition of conditioned medium to P8-sorted DPCs. (B) Edu assay showed a significant increase in proliferation of P8DPC sorted with the addition of conditioned medium. (C) Indicates the proportion of EdU-positive DPCs. (D) Elevated ALP activity in sorted DPC after addition of conditioned medium. (E) The mRNA expression levels of Versican, Sox18, HHIP, BMP4 and LEF1 were significantly increased by the addition of conditioned medium, including Sox18 and HHIP. Data are expressed as mean±SEM (n = 3), with significance determined by unpaired Student’s t-test (* p<0.05; ** p<0.01; *** p<0.001). Scale bars: 200 μm for A and B, and 100 μm for D. DPCs, dermal papilla cells; SEM, standard error of the mean.

With increasing passages, flow-sorted DPCs lost their ability to form spherical agglutinations by the eighth generation, whereas mechanically isolated DPCs retained strong agglutination capability. To test the effect of supportive factors, we added conditioned medium from mechanically isolated DPCs to the culture of eighth-generation flow-sorted DPCs, using regular medium as a control. Results indicated that the conditioned medium promoted spherical agglutination in flow-sorted DPCs (Figure 7A). The EdU assay showed a significant increase in the positivity rate of DPCs with the addition of conditioned medium compared to the control group (Figures 7B, 7C). Similarly, alkaline phosphatase activity increased in cells after the addition of conditioned medium (Figure 7D). Additionally, mRNA levels of genes associated with the induction capacity of DPCs showed a significant increase after the addition of conditioned medium (Figure 7E).

## DISCUSSION

DPCs, are dermal-derived cells located at the base of hair follicles. Originating from dermal mesenchymal differentiation, they condense into spherical structures. DPCs play a crucial role in determining hair follicle type, influencing hair growth, and contributing to hair quality [21]. Studies have confirmed that hair follicles cease to grow in the absence of the dermal papilla [22,23]. Hair follicles can be reconstructed *in vivo* following treatment with primary or early-stage cells [24,25]. Consequently, utilizing DPCs as seed cells to study their properties and functions *in vitro* has become a primary research approach [26,27]. The surface of DPCs is coated not only by the basement membrane but also enveloped by surrounding hair matrix cells and the dermal sheath [28]. This unique spatial location presents challenges for the isolation and culture of DPCs. Consequently, isolating DPCs and establishing reliable *in vivo* culture methods are essential prerequisites for advancing biological research on wool tissue. Initially, DPCs were isolated from human scalp hair follicles through microdissection, with mechanical separation methods commonly applied in studies of other species.

However, there are currently differing views on the morphology of DPCs isolated through microdissection; some describe them as flat and polygonal, while others report an elongated, spindle-like shape [29,30]. This discrepancy complicates the identification of isolated cells as true DPCs. Furthermore, obtaining a sufficient number of characteristic *in vivo* cells for high-throughput analyses, such as RNA-seq and ChIP-seq, remains challenging with the current mechanical separation methods. While certain markers enable flow sorting in mice, these markers are not expressed in ovine cells, limiting their applicability in ovine DPC studies.

Due to the limited research on the transcriptome of ovine DPCs, suitable molecular markers for antibody-based screening have yet to be identified. To address this, based on previous single-cell transcriptome sequencing of ovine dorsal skin [17], we systematically screened the ovine DPC transcriptome for surface markers and employed antibody-based sorting of DPCs in combination with flow cytometry. This approach led to the identification of PDGFRA, a surface protein specifically expressed in DPCs within hair follicle cells.

Using this approach, we successfully sorted 1.91% of PDGFRA^+^ DPCs. By applying the specific marker PDGFRA to label ovine DPCs obtained through both mechanical separation and antibody labeling methods, flow cytometry analysis revealed that DPCs isolated by mechanical separation were mixed with other cell types. In contrast, antibody labeling increased the purity of DPCs to 95%, marking a significant improvement. Looking forward, we plan to apply PDGFRA for flow sorting of DPCs in other species.

However, a limitation of antibody-labeled sorting is the small number of cells obtained before and after sorting. During the preparation of hair follicle single-cell suspensions, the cell count is substantially reduced due to multiple centrifugation and filtration steps, further decreasing the number of sorted DPCs and complicating their direct use in experimental research. To address this, we attempted to increase the yield of single cells by slightly raising centrifugation speed, extending enzyme digestion time, and reducing the frequency of centrifugation and filtration. Additionally, we observed that in early passages, cultured DPCs tend to form multi-layered clusters; however, this clustering tendency diminishes with continued passaging [28]. Our sorted and cultured P3 DPCs still possess this clumping ability. Versican, α-SMA, and ALP are markers for DPCs, and their expression levels can serve as indicators of the purity and properties of DPCs. Our identification of these three markers confirmed that the cells we sorted were indeed DPCs and possessed the properties of DPCs.

Hair follicle morphogenesis results from interactions between epithelial and mesenchymal cells, underscoring the crucial role of cellular communication within the organism. DPCs, hair follicle stem cells, and mesenchymal stem cells, among others, release a variety of cytokines and growth factors, broadly categorized as secretagogues or conditioned media. These secreted factors include cytokines, chemokines, cell adhesion molecules, lipid mediators, interleukins, growth factors, hormones, as well as extracellular vesicles like exosomes and microvesicles [31]. Conditioned mediators secreted through cellular interactions play a vital role in tissue repair and regeneration. Notably, CD34- and Lgr5-positive hair follicle stem cells contribute significantly to this process, given their abilities for self-renewal and pluripotent differentiation [32].

In animal organisms, DPCs naturally aggregate within the dermis to form a three-dimensional multicellular structure, and three-dimensional cultures can better mimic the multilayered structure of DPCs *in vivo* [33]. Therefore, a variety of three-dimensional cultures have been described [34–36]. And this culture mode can indeed maintain or enhance the properties of DPCs.

## CONCLUSION

Our study highlights the significant role of DPCs in hair follicle development and their potential as a model for understanding hair biology. Through the use of flow cytometry and specific markers such as PDGFRA, we successfully isolated and characterized DPCs with high purity. Our findings indicate that while flow-sorted DPCs exhibit certain limitations in long-term culture, the addition of conditioned medium from mechanically isolated DPCs can enhance their viability and functional properties. This work not only underscores the importance of DPCs in regulating hair growth and quality but also lays the groundwork for future research aimed at leveraging these cells for therapeutic applications in hair regeneration and related fields.

## Figures and Tables

**Table 1:** List of antibodies used for immunofluorescence staining

Antibody	Manufacture	Catalog no.
PFGFRA antibody	Abcam	ab203491
PFGFRA antibody	Abcam	ab270086
TMEM119 Monoclonal antibody	Proteintech	66948-1-Ig
Anti-VCAN rabbit polyclonal antibody	Sangon Biotech	D223532
Anti-ACTA1 rabbit polyclonal antibody	Sangon Biotech	D121592
Goat anti-rabbit IgG H&L (Alexa Fluore ®488)	Abcam	Ab150077
Goat anti-rabbit IgG H&L (Alexa Fluore ®555)	Abcam	Ab150078
Goat anti-mouse IgG H&L (Alexa Fluore ®488)	Abcam	Ab150113

**Table 2:** Primers used for qRT-PCR

Gene	Primer sequence (5′-3′)	Product size (bp)
*VCAN*	F:TACAAAGGGAGGGTGTCGGT R: AAGCCTTCTGTGCCATCTCA	226
*LEF1*	F:CAGGTGGTGTTGGACAGATAA R: ATGAGGGATGCCAGTTGTG	179
*Sox18*	F: TGTGGGCGAAGGACGAGC R:GCCAAGCCTGGGAGGAGGAG	253
*BMP4*	F: TAGCAAGAGCGCAGTCATCC R: AGCAGAGTTTTCGCTGGTCC	196
*HHIP*	F: GTGGCCTGTGCTTTCCTGAT R:AGAATGAAGAGGCGGTGGGA	208

qRT-PCR, quantitative reverse transcription polymerase chain reaction.

**Table 3:** Identification of DPCs-specific membrane proteins

Ranking	Gene	Cell localization	p_val_adj	Antibody availability
1	*EIF2S2*	Membrane	0	NO
2	*TMEM119*	Membrane	0	Yes
3	*GPC3*	Membrane	0	Yes
4	*HHIP*	Cytoplasm and Cell membrane	0	NO
5	*CD63*	Membrane	0	Yes
6	*LAMC3*	Membrane. Nucleus. Cytoplasm. Cytoplasmic vesicle	0	NO
7	*PDGFRA*	Membrane	0	Yes
